# Love in the Time of Pyrethroids: Mating Behavior of *Sitophilus zeamais* Is Influenced by Sublethal Concentrations of λ-Cyhalothrin and Lateralization

**DOI:** 10.3390/insects16080865

**Published:** 2025-08-20

**Authors:** Maria C. Boukouvala, Nickolas G. Kavallieratos, Demeter Lorentha S. Gidari, Constantin S. Filintas, Anna Skourti, Vasiliki Panagiota C. Kyrpislidi, Dionysios P. Skordos

**Affiliations:** Laboratory of Agricultural Zoology and Entomology, Faculty of Crop Science, Agricultural University of Athens, 75 Iera Odos Str., 11855 Athens, Greece; mbouk@aua.gr (M.C.B.); dlgidari@aua.gr (D.L.S.G.); kfilintas@aua.gr (C.S.F.); annaskourti@aua.gr (A.S.); vassokyrp@gmail.com (V.P.C.K.); dionsko2@gmail.com (D.P.S.)

**Keywords:** maize weevil, low insecticidal concentrations, pyrethroid insecticide, stored grain pest, copulation success

## Abstract

*Sitophilus zeamais* Motschulsky (Coleoptera: Curculionidae) is one of the most destructive pests of stored grains worldwide. Sublethal concentrations of insecticides are known to influence insect behavior, potentially disrupting critical processes such as mating. This study investigated the effects of λ-cyhalothrin at the lethal concentration (LC) values LC_10_ and LC_30_ and lateralization on the mating behavior patterns of *S. zeamais* males. Results showed that the exposure to sublethal concentrations of λ-cyhalothrin significantly altered the copulation success rate and key time-related parameters, including mate recognition and copulation duration, while the lateralization caused significant differences in mating time-related parameters within each tested group (control, LC_10,_ and LC_30_). Additionally, the λ-cyhalothrin-treated groups showed prolonged mate recognition times and required more mounting attempts to achieve mating. These findings highlight the potential of sublethal insecticide applications to control *S. zeamais* populations by impairing reproduction.

## Simple Summary

The present study investigates the alterations of the mating behavioral traits of *Sitophilus zeamais* under sublethal concentrations of the pyrethroid λ-cyhalothrin and the impact of lateralization. The treated individuals demonstrated altered behavior compared to the untreated individuals. Significant changes were observed in the mating success rates, the mate recognition time, and the duration of the copulation, among the tested groups (control, LC_10_, and LC_30_). The lateralization significantly impacted mating behavioral traits, such as the interaction between the males and females and the duration of the copulation. These results suggest that low concentrations of λ-cyhalothrin might disrupt the reproduction of *S. zeamais*, offering a new angle for pest control strategies that focus on behavioral alterations.

## 1. Introduction

*Sitophilus zeamais* Motschulsky (Coleoptera: Curculionidae) is a noxious insect of stored grains worldwide, particularly in tropical and subtropical regions [1,2,3,4]. It is a primary pest of maize [5,6] but is also known to infest other grains such as wheat, rice, and sorghum [7,8,9]. In addition to direct consumption of the grains, *S. zeamais* infestation leads to increased moisture content and fungal contamination, further compounding post-harvest losses [10,11].

The type II pyrethroid λ-cyhalothrin is an efficient insecticidal agent against various insect species, including stored-product pests [12,13,14,15,16]. It interacts with neuronal membrane sodium (Na^+^) channels, which are essential for the response of the insect to nerve inputs. When λ-cyhalothrin reaches the active site, it causes uncontrollable nerve discharges, potentially leading to tremors, twitches, and paralysis [17]. It can also interact with the calcium and chloride channels, quickly impairing muscular control and feeding disruptions, as it is easily absorbed by biological tissues and penetrates the cuticle of the insect [18]. λ-Cyhalothrin has also been tested for the effect of the sublethal concentrations on numerous insect species [19,20,21,22]. Under the influence of sublethal insecticidal concentrations, insects often exhibit various behavioral alterations, including locomotion, selection of oviposition sites, mating behavior and copulation success, and disruptions in the chemical communication systems [21,23,24,25].

Lateralization (i.e., asymmetries in the two hemispheres of the brain and behavior) has been thoroughly investigated in vertebrates, ants, and honeybees [26,27,28,29,30,31]. In recent years, various studies have explored the lateralization of the mating behavior of stored product pests [24,32,33,34].

The rapid population growth of *S. zeamais* favors the extensive damage of the infested commodities [35,36,37,38]. It has been previously documented that insecticides at sublethal concentrations compromise the development of stored-product insects [39,40]. However, there is no data about the effect of λ-cyhalothrin at low concentrations on the mating behavior of *S. zeamais*. Thus, the current study aims to investigate the impact of λ-cyhalothrin LC_10_ and LC_30_ on the courtship and mating of *S. zeamais*, taking into account the influence of males’ lateralization.

## 2. Materials and Methods

### 2.1. Sitophilus zeamais Colonies and Sex Recognition

The male and female *S. zeamais* adults that were used in the bioassays were obtained from the Laboratory of Agricultural Zoology and Entomology (Athens, Greece). *Sitophilus zeamais* individuals were reared on insecticide-free maize in devoid of infestation or impurities [41]. The colonies were maintained in controlled conditions (65% relative humidity (RH) and 30 °C) and continuous darkness [21,42]. One day, newly emerged adults, obtained from separately kept maize kernels, were sexed according to Halstead [43] and Tolpo and Morrison [44], based on the pattern of their rostrum punctuation, length, and texture. Then they were kept separately, by sex, in jars with rearing medium until sexual maturation (4 days old) [36]. The female individuals were marked with white color on their thorax to allow sex recognition during the bioassays.

### 2.2. Insecticide

The commercial formulation ICON 10 CS (Syngenta, Anthousa, Greece), with λ-cyhalothrin as the active ingredient (a.i.) at 10.7 *w*/*w,* was used for the bioassays.

### 2.3. Contact Toxicity Bioassays

To determine the LC_10_, LC_30_, and LC_50_ of λ-cyhalothrin against male and female adults of *S. zeamais*, six dilutions (i.e., 0.0025, 0.0013, 0.0008, 0.000625, 0.0005, and 0.00042 mg a.i./cm^2^) were prepared in distilled water. Then, separate filter papers (Whatman No. 1) cut into a circular shape and placed at the bottom of a Petri dish (50.27 cm^2^) were impregnated, using a micropipette, with 1 mL of the various dilutions and left to dry at 30 °C for 2 h. Control filter papers were impregnated with 1 mL of distilled water. After the filter papers had dried, 20 *S. zeamais* individuals were placed in each dish (6 concentrations × 5 replications = 30 dishes). Then the dishes were maintained in 65% RH, 30 °C, and continuous darkness for 72 h, and the total number of dead adults was counted. Prior to the behavioral bioassays, new male and female *S. zeamais* individuals were exposed to the LC_10_, LC_30_ of λ-cyhalothrin or the control, as described above.

### 2.4. Mating Behavior Bioassays

The experiment was conducted in a Petri dish arena surrounded by a filter paper box to prevent any disruption of the individuals from the observer or the surrounding area [32]. The walls of the dishes were covered with liquid polytetrafluoroethylene (Sigma-Aldrich Chemie GmbH, Taufkirchen, Germany) to prevent insects’ escapes. Prior to each observation, *S. zeamais* individuals were adjusted to the natural conditions of light for a 3 h interval [24,33,34,45]. A male and a female *S. zeamais* (virgins), treated with LC_10_ or LC_30_ of λ-cyhalothrin or the control, were placed in the arena and were visually recorded for 60 min (min) or until the end of the mating process [46]. For each mating pair, the following parameters were recorded: (1) the side (back, left, right or front) of the body of the female that the male approached, (2) the mounted side (back, left, right or front) of the body of the female, (3) the mounting attempts of males (number), (4) the stroking behavior, if any, (5) the copulation success, if any, and (6) the side (back, left, right or front) of leaving (which side the male leaves the body of the female). The courtship time parameters were observed for (1) the duration of mate recognition, and (2) the duration of the interaction between the two individuals, mounting attempts of males, and the walking that the individuals performed attached, consisting of precopula. The duration of the copulation was also recorded. When 50 successful mating pairs were observed per treatment or control, counting was completed. Thus, 90, 131, and 166 pairs were recorded in total for control, LC_10_, and LC_30_, respectively. However, pairs where females stayed next to the arena walls, affecting the males’ approach choice, and pairs that were motionless or did not engage in any sexual contact were rejected [33]. Therefore, 13, 20, and 27 pairs were removed from the control, LC_10_, and LC_30_ groups, respectively. As a result, there were 77 (control), 111 (LC_10_), and 139 (LC_30_) mating pairs included in the statistical analysis.

### 2.5. Statistical Analysis

LC_10_, LC_30,_ and LC_50_ of λ-cyhalothrin were estimated using probit analysis by the R software (version 2.15.1), with a 95% confidence interval (CI) [47,48]. Data on the laterality behavior was analyzed using the software JMP 16.2 [49]. The courtship time parameters, which did not follow a normal distribution, were analyzed using the Steel–Dwass test with a significance level of 0.05 [45]. The mating success was calculated by a weighted generalized linear model with binomial distribution: *y* = *Xβ* + *ε* (*y*: the vector of observations, i.e., successful or not successful copulation; *X*: the incidence matrix; *β*: the vector of fixed effect, i.e., λ-cyhalothrin LC_10_-, LC_30_-exposed, and control; *ε*: the vector of the random residual effect). To detect the significant differences between values, the 0.05 level of probability was used.

## 3. Results

### 3.1. Contact Toxicity of λ-Cyhalothrin on S. zeamais Adults

The bioassays on the contact toxicity of λ-cyhalothrin on *S. zeamais* adults are presented in Table 1.

### 3.2. Impact of λ-Cyhalothrin and Laterality on the Mating Success of S. zeamais

**Figure 1:**
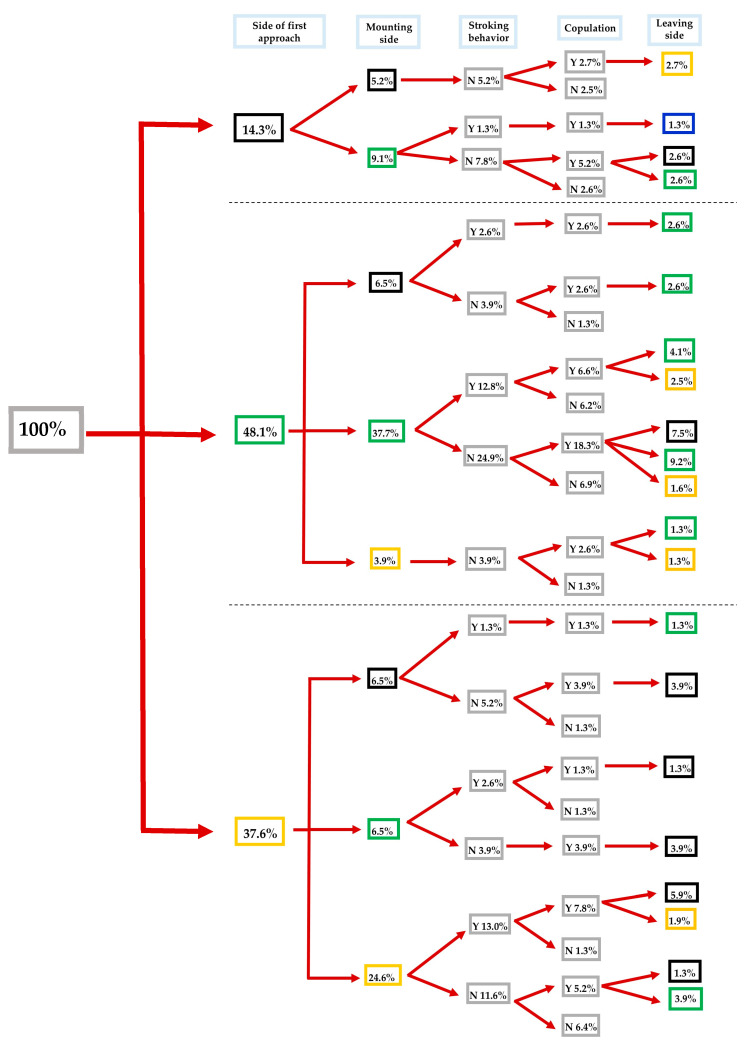
Flow chart of courtship and mating behavior of *Sitophilus zeamais* adults exposed to control. Different lateralized traits (i.e., approaching, mounting, and leaving the female’s body) exhibited by males during each behavioral phase are indicated by the color of the boxes: black, green, yellow, and blue for back, left, right, and front side, respectively. Inside each grey box: Y = Yes and N = No (*n* = 77 pairs).

Most of the control males showed a left-biased approach tendency (48.1 out of 100%), compared to right and back (37.6 and 14.3 out of 100%, respectively). The majority of the left-biased control males mounted their mate from their left side (37.7% out of 48.1%). The 12.8 out of 37.7% left-biased males performed stroking behavior, of which 6.6% (out of 12.8%) mated successfully. The 24.9% out of 37.7% did not perform stroking behavior, where 18.3% out of 24.9% achieved successful copulations and left the body of the females from their back, left, and right sides at rates of 7.5, 9.2, and 1.6%, respectively (Figure 1).

**Figure 2:**
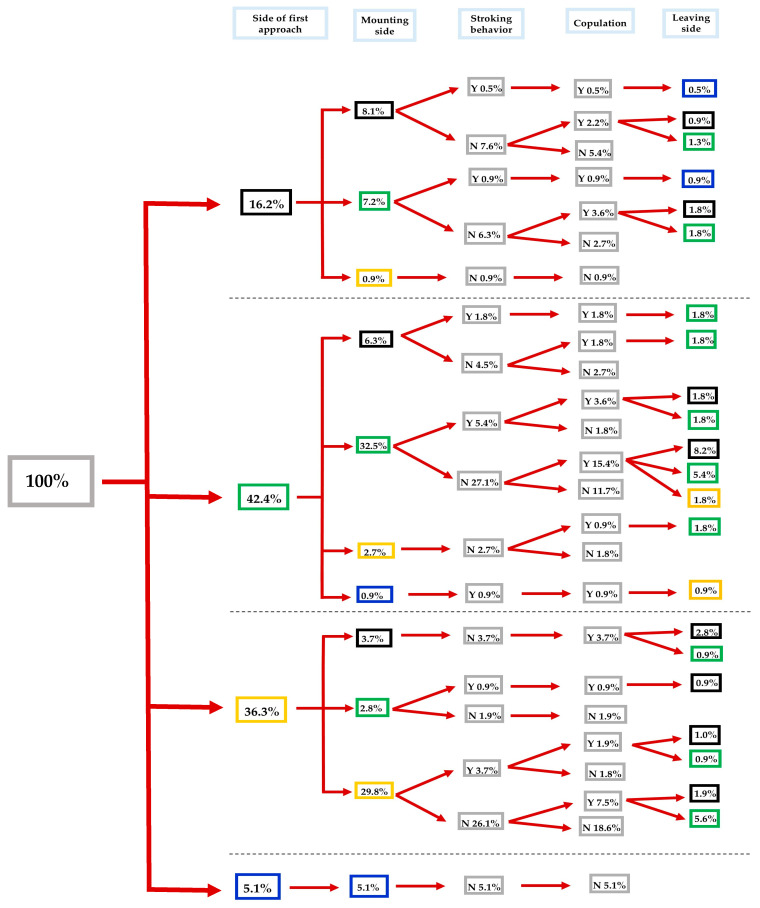
Flow chart of courtship and mating behavior of *Sitophilus zeamais* adults exposed to λ-cyhalothrin LC_10_. Different lateralized traits (i.e., approaching, mounting, and leaving the female’s body) exhibited by males during each behavioral phase are indicated by the color of the boxes: black, green, yellow, and blue for back, left, right, and front side, respectively. Inside each grey box: Y = Yes and N = No (*n* = 111 pairs).

Concerning the λ-cyhalothrin LC_10_-treated *S. zeamais* individuals, most of the males had a left-biased tendency (42.4 out of 100%), compared to the other directions (36.3, 16.2, and 5.1 out of 100% for right-biased, back-side, and front-side, respectively). The 32.5 out of 42.4% of the left-biased males tend to mount the females from their left side. Most of them, 27.1 out of 32.5%, did not perform stroking behavior, where 15.4 out of 27.1% of these males achieved successful copulations, leaving the body of the female from their back, left, and right sides at rates of 8.2, 5.4, and 1.8%, respectively. In the case of right-biased approaches (36.3%), most of the males performed mountings from the right side of the female’s body (29.8 out of 36.3%), with 26.1 out of 29.8% of them not showing stroking behavior, and finally, 7.5% (out of 26.1%) mated with success (Figure 2).

**Figure 3:**
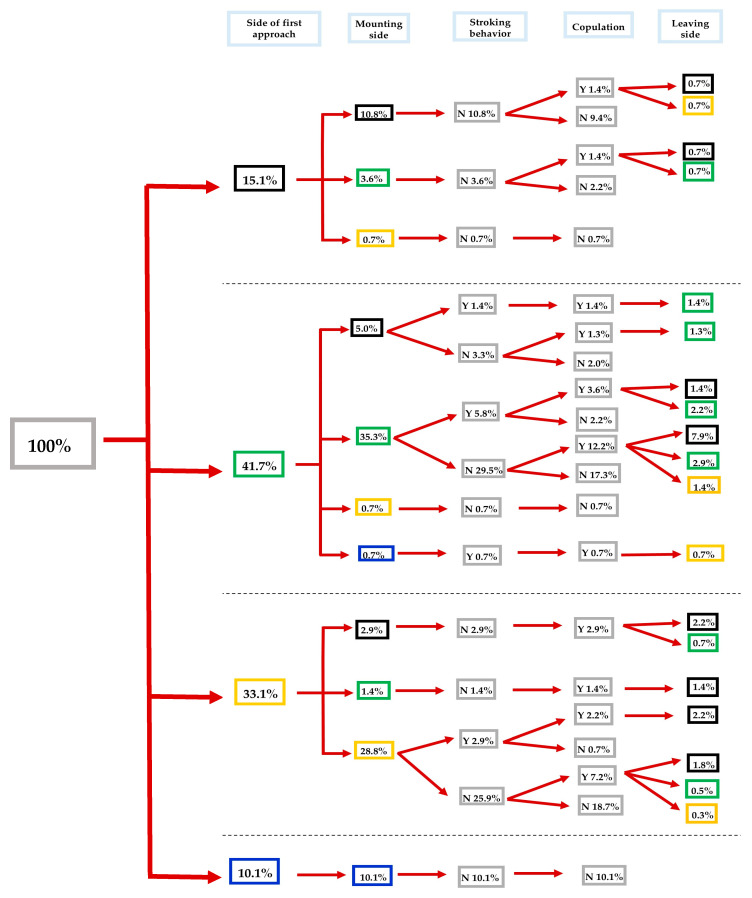
Flow chart of courtship and mating behavior of *Sitophilus zeamais* adults exposed to λ-cyhalothrin LC_30_. Different lateralized traits (i.e., approaching, mounting, and leaving the female’s body) exhibited by males during each behavioral phase are indicated by the color of the boxes: black, green, yellow, and blue for back, left, right, and front side, respectively. Inside each grey box: Y = Yes and N = No (*n* = 139 pairs).

A similar pattern, as in the control and the λ-cyhalothrin LC_10_ groups, was recorded for the λ-cyhalothrin LC_30_-treated *S. zeamais* male individuals, showing a left-biased tendency for the approach (41.7 out of 100%), compared to right-biased, back-side, and front-side (33.1, 15.1, and 10.1 out of 100%, respectively). Most of the left-biased λ-cyhalothrin LC_30_-treated males mounted the females from their left side (35.3 out of 41.7%). The 29.5 out of 35.3% did not perform stroking behavior, where 12.2 out of 29.5% of males had successful copulation and 17.3 out of 29.5% failed to mate. The majority of males that demonstrated right-biased approaches mounted females from their right side (28.8 out of 33.1%), did not perform the stroking behavior (25.9 out of 28.8%), while only 7.2 out of 25.9% succeeded in copulation (Figure 3).

**Figure 4:**
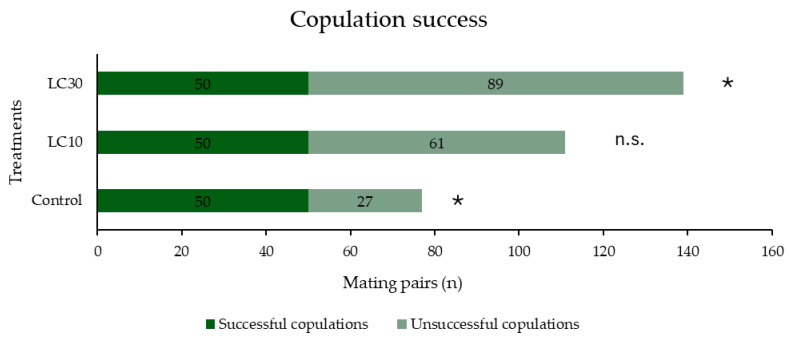
Copulation success of *Sitophilus zeamais* adults exposed to control, or LC_10_ or LC_30_ of λ-cyhalothrin. The asterisks indicate significant differences (generalized linear model, binomial distribution, *p* < 0.01), n.s. = not significant.

In Figure 4, the mating success of the treated (LC_10_, LC_30_), and control *S. zeamais* is presented. Fifty mating couples of each tested group achieved successful copulations. The successful copulation of males was affected by their exposure to LC_10_ and LC_30_ of λ-cyhalothrin (*χ*^2^ = 29.0, df = 2, *p* < 0.01). Mating success of *S. zeamais* males exposed to control and LC_30_ of λ-cyhalothrin was significantly affected (*χ*^2^ = 6.9, df = 1, *p* > 0.01 and *χ*^2^ = 11.0, df = 1, *p* > 0.01, respectively). Mating success of λ-cyhalothrin LC_10_-treated males was not affected significantly (*χ*^2^ = 1.1, df = 1, *p* > 0.05) (Figure 4).

### 3.3. Impact of λ-Cyhalothrin and Laterality on S. zeamais Mating Time Parameters

#### 3.3.1. Impact of the Direction of the Approach

The side that males approached the females affected the mate recognition time. Within each tested group (control, λ-cyhalothrin LC_10_, and λ-cyhalothrin LC_30_), there were significant differences among the various directions. Significant differences were also observed among the tested groups. The λ-cyhalothrin LC_30_-treated males, which approached their mates from their back side, performed a significantly longer time interval to detect their mates (3.9 min), compared to all the other males of the three tested groups (control, λ-cyhalothrin LC_10_, and λ-cyhalothrin LC_30_). The significantly shortest mate recognition time was recorded for the left-biased control males (1.8 min), compared to all the other males of the different directions and tested groups (Table 2).

The intervals of the interaction between the two individuals (female and male) were significantly influenced by the direction of the approach and the insecticidal treatment. The left-biased control males spent significantly the shortest time (3.8 min) interacting with the females, compared to those of the control group that approached from the back and right sides, as well as compared to the males of all directions of the two λ-cyhalothrin-treated groups (Table 2).

The control males that first approached their mates from their back side performed significantly longer walking time (7.2 min), compared to all the other males (left- and right-biased control males, males of the λ-cyhalothrin LC_10_ and LC_30_ groups that first approached their mates from their back, left, right, or front side) (Table 2).

Concerning the mounting attempts, control males performed significantly fewer attempts to mount their mates (1.6 and 1.7 times for the left- or right- and for the back- side approaching males, respectively), compared to the λ-cyhalothrin LC_10_ or the λ-cyhalothrin LC_30_ males. λ-Cyhalothrin LC_30_ males that first approached their mates from their front side performed significantly more mounting attempts, vs. all the other males, except for the front-approaching males of the λ-cyhalothrin LC_10_ group (Table 2).

Regarding the duration of the copulation, males of all directions of the control group mated for significantly longer times (276.9, 274.1, and 268.1 min, for the left-, right-, and back-side approaching males), compared to the males of all directions of the two λ-cyhalothrin-treated groups. The front-side approaching males of the λ-cyhalothrin LC_30_ group copulated for significantly shorter time (18.6 min), compared to all the other males of all directions and the tested group (Table 2).

#### 3.3.2. Impact of the Mounting Direction

The mate recognition time was not significantly affected by the laterality within the control group; however, it was significantly increased in the λ-cyhalothrin-treated groups, compared to the control. The significantly shortest mate recognition times were recorded for the control males (2.1, 2.0, and 1.9 min for the back-, right-, and left-mounting males). The longest time (3.9 min) was detected for the λ-cyhalothrin LC_30_-treated males that mounted their mates from their back side, compared to all the other males, except for the front-mounting males of the two λ-cyhalothrin groups (Table 3).

The time of the interaction was significantly longer for the λ-cyhalothrin LC_30_ males that mounted their mates from their front side (9.9 min), compared to all the other males of all directions of the three tested groups (control, λ-cyhalothrin LC_10_, and λ-cyhalothrin LC_30_), except for the front-mounting males of the λ-cyhalothrin LC_10_ group (8.9 min). In contrast, the shortest interaction time (4.5 min) was recorded at the left-mounting males of the control group, significantly shorter compared to all the other males (Table 3).

The walking time was not significantly influenced by the lateralization within each tested group (control, λ-cyhalothrin LC_10_, and λ-cyhalothrin LC_30_) but was significantly influenced by the insecticidal application. The longest walking periods were detected for the control males (5.3 and 5.2 min for the back- or left- and right-mounting males, respectively), which were significantly different from the λ-cyhalothrin LC_10_- and λ-cyhalothrin LC_30_-treated males (Table 3).

The number of mounting attempts for the control males of all directions was significantly shorter (1.7 and 1.6 times for the back- or left- and right-mounting males, respectively), compared to all the males of the λ-cyhalothrin-treated groups (Table 3).

The application of the insecticide and the laterality significantly affected the duration of the copulation of each tested group (control, λ-cyhalothrin LC_10_, and λ-cyhalothrin LC_30_). The use of λ-cyhalothrin LC_10_ and LC_30_ caused a significant decrease in the copulation time, compared to the control group. The right-mounting males of the control group copulated for significantly longer time (309.7 min), compared to all the other males of all directions and tested groups. The front-mounting males of the λ-cyhalothrin LC_10_ group and the right- and front-mounting males of the λ-cyhalothrin LC_30_ group performed the shortest durations of copulation (61.8, 51.8, and 44.6 min, respectively) (Table 3).

## 4. Discussion

The current study demonstrates that λ-cyhalothrin, even at low concentrations, radically alters key aspects of mating interactions, including copulation success, and time-related parameters such as mate recognition time and copulation duration. The observed alterations in the mating traits at both λ-cyhalothrin-treated groups (LC_10_ and LC_30_) suggest neurophysiological interference driven by the insecticidal mode of action on the neural ion channels of the insects [17,18], given that the behavioral traits refer to the function of the brain [50]. The influence of the insecticidal action proved decisive for the success of copulation. The present study documents that the overall successful copulation rate followed a decreasing order among treatments, i.e., LC_30_ (41.2%) < LC_10_ (51.6%) < control (65.3%). Similarly, when *Alphitobius diaperinus* (Panzer) (Coleoptera: Tenebrionidae) adults were exposed to sublethal concentrations of another pyrethroid insecticide, α-cypermethrin, the successful copulation rates were 87.2, 63.4, and 64.4% for the control, LC_10,_ and LC_30_ groups, respectively [24].

Here, the mate detection time was significantly increased in the insecticide-treated groups. This pattern has also been observed in other studies. *Prostephanus truncatus* (Horn) (Coleoptera: Bostrychidae) adults exposed to sublethal concentrations of *Acmella oleracea* (L.) R.K. Jansen (Asteraceae) hexane extract resulted in prolonged mate recognition duration [51]. Additionally, *A. diaperinus* α-cypermethrin LC_10_- and LC_30_-treated adults resulted in increased mate detection duration [24]. The number of mounting attempts was also notably higher in all the side tendences of the λ-cyhalothrin LC_10_ and LC_30_ groups, vs. the control. Given that the mounting is crucial for the mating of *S. zeamais* [46,52], the predicament of this behavioral trait could prevent the achievement of successful copulation. Insects depend on mating as a means of population growth, establishment in new habitats, and coexistence with other species [53]. Since the difficulty or delay of mate recognition, decreased copulation time, and mating success rates have been proven to lead to the inability of the population growth [54,55], the sublethal concentrations of λ-cyhalothrin could decrease the population of *S. zeamais*.

*Sitophilus zeamais* females create holes in the kernel, where they lay eggs and then seal the holes using a secretion [56,57]. Larvae borrow the internal part of the kernels, which contain carbohydrates and other nutrients, causing severe weight loss [57,58]. Given that the development until the adult stage takes place inside the kernel, the detection of the infestation and the control of this species is challenging [59]. Thus, it is important to target the management of *S. zeamais* adults. Assuming that the negative effect of the sublethal concentrations of λ-cyhalothrin on the mating behavior and success of *S. zeamais* could prevent the fecundity and the oviposition process, the current treatment could eliminate the qualitative and quantitative damage of the maize kernels. Interestingly, a recent study on another coleopteran species demonstrated that sublethal concentrations of λ-cyhalothrin significantly reduced the average fecundity and prolonged the durations of all the developmental stages of *Henosepilachna vigintioctomaculata* (Coccinellidae) [16]. However, this issue needs to be further investigated.

Left-biased males of all three tested groups achieved higher copulation rates compared to those of the other sides (back, right, and front). This pattern is common among various coleopteran families. For instance, it has been evident that different tenebrionids tend to achieve higher rates of successful copulations when they demonstrate a left-biased tendency [33,34]. The exposure of *S. zeamais* adults to λ-cyhalothrin did not affect the tendency of the side of the approach. Similarly, *A. diaperinus* males and females, that had been treated with α-cypermethrin LC_10_ and LC_30_, retained their right-biased tendency approaching their mates, as in the control group [24]. Furthermore, when Romano et al. [32] tested the impact of the geographical origin and the rearing medium of *Sitophilus oryzae* (L.) (Coleoptera: Curculionidae), the left-biased copulation tendency, which led to higher mating success, was not affected for the adults of all three strains (two Greek strains, one reared on wheat and one on maize, and a Peruvian strain reared on maize). The maintenance of specific lateral tendencies under the influence of insecticidal agents or environmental parameters indicates that they are strongly stable. Whether they can be altered under stronger influences, such as higher insecticidal concentrations, needs further research.

## 5. Conclusions

The results of the current study highlight the importance of incorporating behavioral traits into the assessment of sublethal insecticidal impacts. Sublethal concentrations of λ-cyhalothrin significantly disrupted the mating behavior of *S. zeamais*, introducing one other component in the concept of management strategies of this species. Nevertheless, future studies employing metabolomics, i.e., studying the changes in the metabolic paths, could shed further light on how λ-cyhalothrin alters the mating traits in stored-product insects.

## Figures and Tables

**Table 1:** Contact toxicity of λ-cyhalothrin on *Sitophilus zeamais* adults after 72 h of exposure.

Active Ingredient	Unit	LC_10_ (95% CI)	LC_30_ (95% CI)	LC_50_ (95% CI)	*χ*^2^ (df = 23)	*p*
λ-cyhalothrin	mg a.i./cm^2^	0.000212 (0.000177–0.000241)	0.000282 (0.000250–0.000308)	0.000344 (0.000317–0.000366)	30.3	1.00

LC = lethal concentration that kills 10%, 30% and 50% of the exposed individuals. 95% CI = lower and upper limits of the 95% confidence interval.

**Table 2:** Effect of the approaching side on the main mating traits of *Sitophilus zeamais* exposed to control, or LC_10_ or LC_30_ of λ-cyhalothrin.

		Precopula					
	Traits	Direction of Approach	Mate Recognition (min)	Interaction (min)	Walking (min)	Mounting Attempts (Number)	Copulation (min)
Treatment							
control		Back	2.8 ± 0.1 D	6.7 ± 0.5 BC	7.2 ± 1.2 A	1.7 ± 0.2 C	268.1 ± 13.6 A
		Left	1.8 ± 0.1 F	3.8 ± 1.0 D	4.8 ± 0.3 B	1.6 ± 0.1 C	276.9 ± 13.9 A
		Right	2.2 ± 0.1 E	6.2 ± 0.6 C	5.1 ± 0.4 B	1.6 ± 0.1 C	274.1 ± 14.2 A
		Tested beetles (number = back + left + right)	11 + 37 + 29 = 77	11 + 37 + 29 = 77	11 + 37 + 29 = 77	11 + 37 + 29 = 77	7 + 25 + 18 = 50
LC_10_		Back	2.8 ± 0.2 CD	7.2 ± 0.6 BC	3.6 ± 0.3 D	4.2 ± 0.8 B	109.2 ± 28.0 C
		Left	2.7 ± 0.1 D	5.9 ± 0.4 C	4.2 ± 0.2 C	3.9 ± 0.5 B	160.4 ± 19.2 B
		Right	3.0 ± 0.1 C	6.5 ± 0.5 C	3.7 ± 0.2 D	3.9 ± 0.5 B	82.4 ± 21.4 C
		Front	3.4 ± 0.3 BC	8.9 ± 1.2 AB	4.1 ± 0.4 BCD	5.8 ± 1.2 AB	46.8 ± 6.7 D
		Tested beetles (number = back+ left + right + front)	18 + 47 + 39 + 7 = 111	18 + 47 + 39 + 7 = 111	18 + 47 + 39 + 7 = 111	18 + 47 + 39 + 7 = 111	6 + 27 + 10 + 7= 50
LC_30_		Back	3.9 ± 0.2 A	8.5 ± 0.6 AB	3.1 ± 0.2 D	4.8 ± 0.6 B	95.1 ± 24.2 C
		Left	3.5 ± 0.1 B	6.2 ± 0.4 C	3.7 ± 0.2 D	4.6 ± 0.5 B	119.1 ± 17.4 C
		Right	3.5 ± 0.1 B	7.6 ± 0.5 B	3.3 ± 0.1 D	4.1 ± 0.5 B	80.4 ± 19.1 C
		Front	3.7 ± 0.2 B	9.7 ± 0.7 A	3.32 ± 0.3 D	6.5 ± 0.6 A	18.6 ± 18.6 E
		Tested beetles (number = back+ left + right + front)	21 + 58 + 46 + 14 = 139	21 + 58 + 46 + 14 = 139	21 + 58 + 46 + 14 = 139	21 + 58 + 46 + 14 = 139	4 + 27 + 18 + 1 = 50
		*χ*^2^, df, *p*	170.0, 10, <0.01	81.6, 10, <0.01	63.7, 10, <0.01	96.7, 10, <0.01	140.5, 10, <0.01

Values are means (±standard errors). Per trait, within each column, different letters indicate significant differences (Steel–Dwass test, *p* < 0.05).

**Table 3:** Effect of the mounting side on the main mating traits of *Sitophilus zeamais* exposed to control, or LC_10,_ or LC_30_ of λ-cyhalothrin.

		Precopula					
	Traits	Direction of Mounting Side	Mate Recognition (min)	Interaction (min)	Walking (min)	Mounting Attempts (Number)	Copulation (min)
Treatment							
control		Back	2.1 ± 0.2 E	5.4 ± 0.7 C	5.3 ± 0.6 A	1.7 ± 0.2 D	248.4 ± 10.8 B
		Left	1.9 ± 0.1 E	4.5 ± 0.3 D	5.3 ± 0.4 A	1.7 ± 0.1 D	269.0 ± 10.1 B
		Right	2.0 ± 0.2 E	6.2 ± 0.8 BC	5.2 ± 0.5 A	1.6 ± 0.1 D	309.7 ± 24.1 A
		Tested beetles (number = back + left + right)	11 + 37 + 29 = 77	11 + 37 + 29 = 77	11 + 37 + 29 = 77	11 + 37 + 29 = 77	7 + 25 + 18 = 50
LC_10_		Back	2.8 ± 0.2 CD	7.2 ± 0.6 BC	3.8 ± 0.3 BC	4.4 ± 1.0 B	115.5 ± 8.2 D
		Left	2.7 ± 0.1 D	5.9 ± 0.4 C	4.1 ± 0.3 B	3.6 ± 0.5 C	133.9 ± 5.3 C
		Right	3.0 ± 0.1 C	6.5 ± 0.5 BC	3.6 ± 0.2 BC	4.1 ± 0.6 C	103.2 ± 5.0 D
		Front	3.4 ± 0.3 ABC	8.9 ± 1.2 AB	4.1 ± 0.4 B	5.3 ± 1.1 AB	61.8 ± 14.9 E
		Tested beetles (number = back+ left + right + front)	20 + 47 + 36 + 8 = 111	20 + 47 + 36 + 8 = 111	20 + 47 + 36 + 8 = 111	20 + 47 + 36 + 8 = 111	9 + 29 + 11 + 1 = 50
LC_30_		Back	3.9 ± 0.2 A	7.8 ± 0.5 B	3.3 ± 0.2 C	4.8 ± 0.6 B	104.3 ± 6.8 D
		Left	3.5 ± 0.1 B	6.2 ± 0.4 C	3.6 ± 0.2 BC	4.6 ± 0.5 B	121.0 ± 2.0 D
		Right	3.5 ± 0.1 B	7.6 ± 0.6 B	3.3 ± 0.1 C	4.1 ± 0.5 BC	51.8 ± 6.7 E
		Front	3.7 ± 0.2 AB	9.9 ± 0.7 A	3.3 ± 0.3 C	6.5 ± 0.6 A	44.6 ± 26.0 E
		Tested beetles (number = back+ left + right + front)	26 + 56 + 42 + 15 = 139	26 + 56 + 42 + 15 = 139	26 + 56 + 42 + 15 = 139	26 + 56 + 42 + 15 = 139	10 + 26 + 13 + 1 = 50
		*χ*^2^, df, *p*	170.5, 10, <0.01	59.5, 10, <0.01	59.4, 10, <0.01	126.8, 10, <0.01	129.6, 10, <0.01

Values are means (±standard errors). Per trait, within each column, different letters indicate significant differences (Steel-Dwass test, *p* < 0.05).

## Data Availability

Data is available within the article.
